# Supplementary material for: CoronaHiT: high-throughput sequencing of SARS-CoV-2 genomes
Source: Genome Med. 2021 Feb 9;13:21. doi: 10.1186/s13073-021-00839-5 (PMC7871948; doi:10.1186/s13073-021-00839-5)

**Fig S1-S6**: Overview of SARS-CoV-2 genome coverage drop-out regions (Ns) of each sample that had a known Ct. Black indicates a region where there is missing data, or where the coverage dropped below 20X for CoronaHiT and ARTIC ONT and 10X for Illumina.

**Fig. S1 -** ARTIC LoCost for routine samples
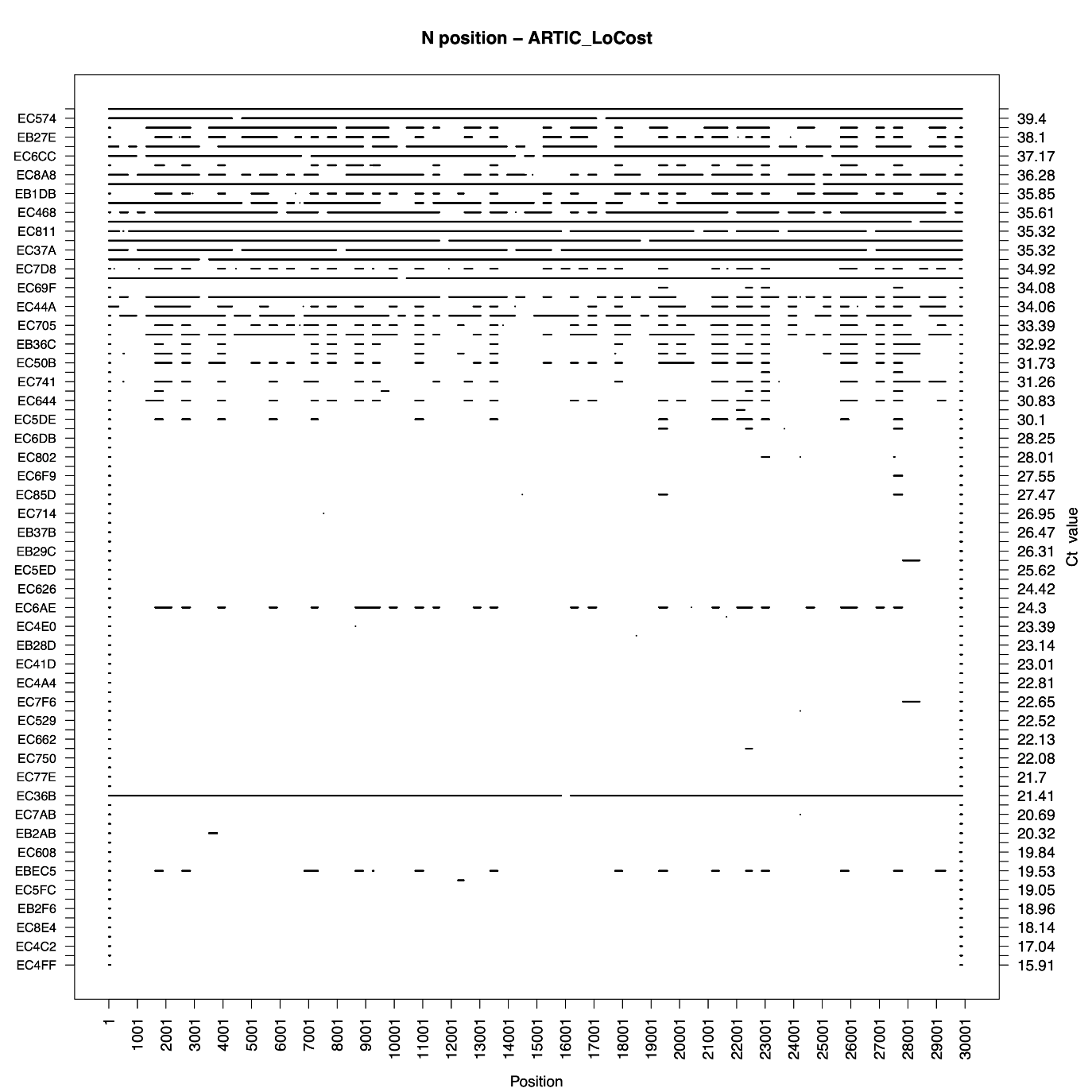


**Fig. S2 -** CoronaHiT-ONT for routine samples **
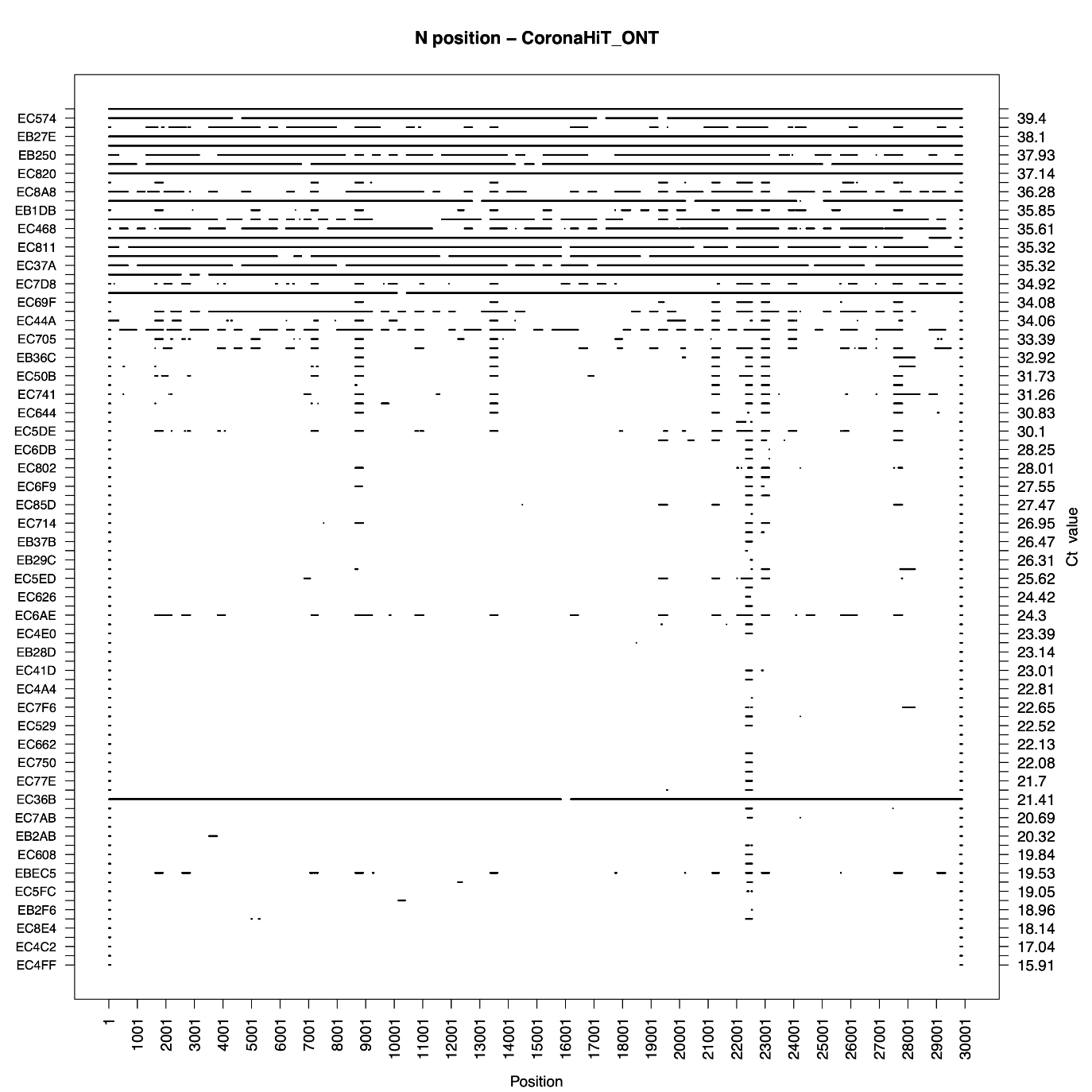
**

**Fig. S3** - CoronaHiT-Illumina for routine samples
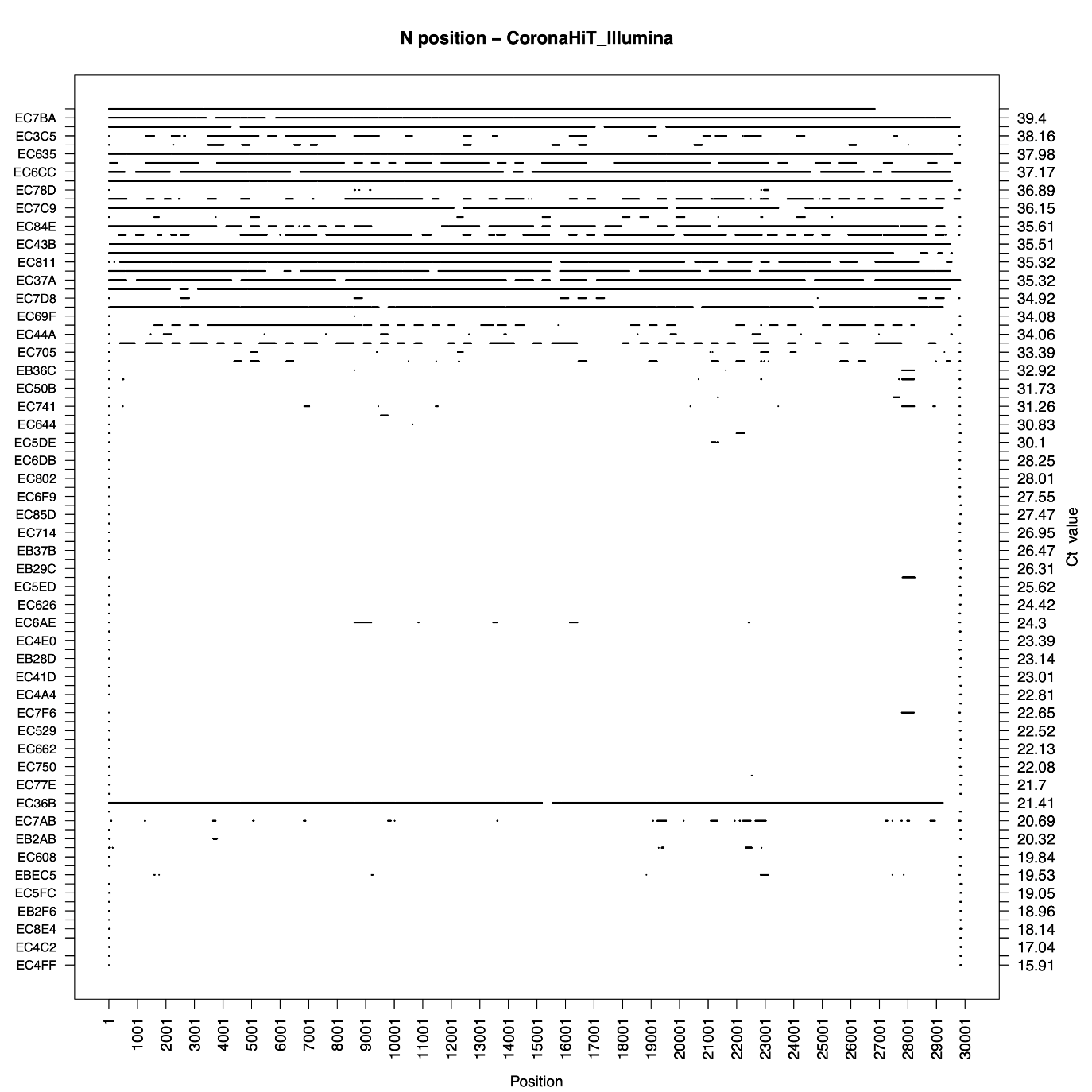


**Fig. S4** - ARTIC LoCost for rapid response samples
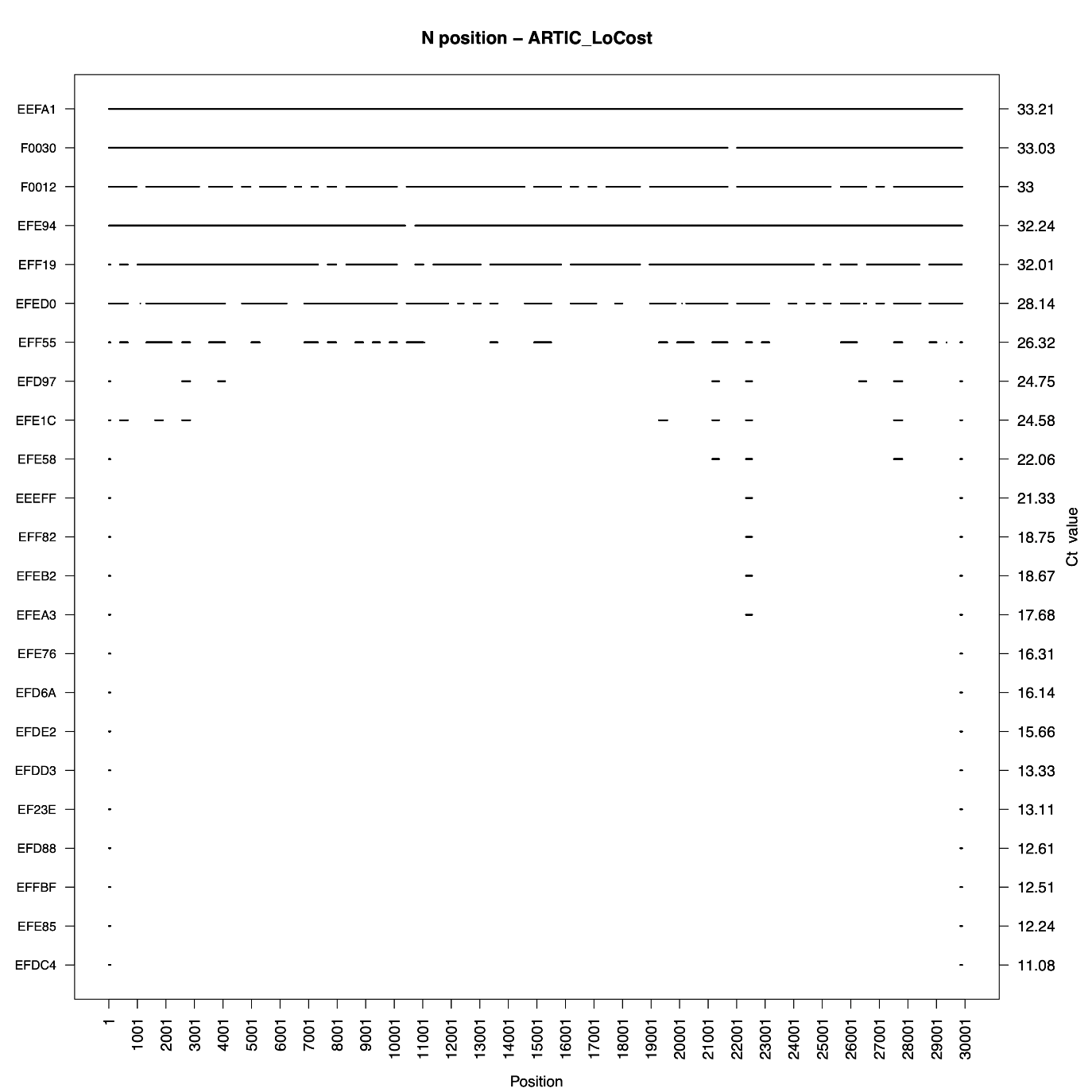


**Fig. S5** - CoronaHiT-ONT for rapid response samples
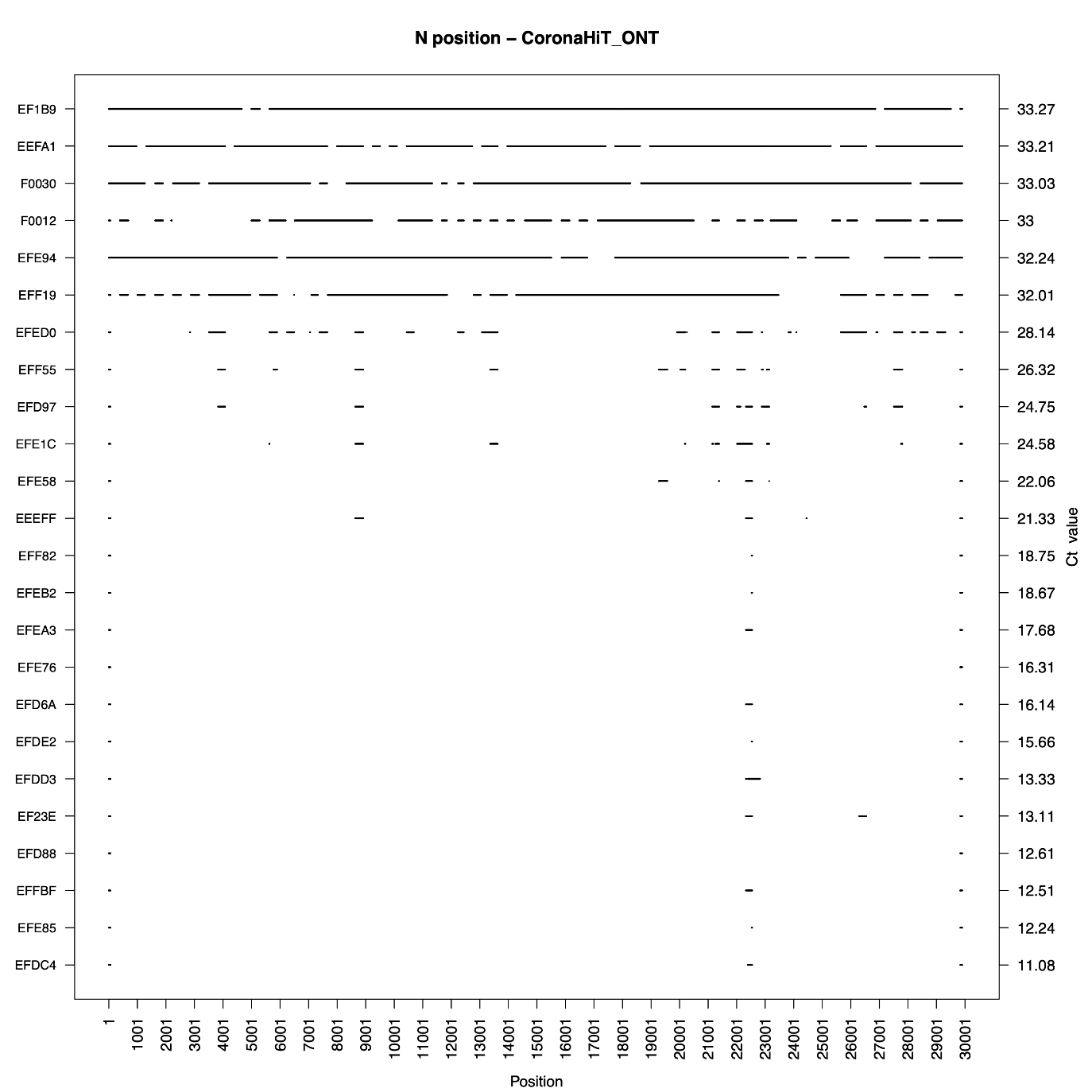


**Fig. S6** - CoronaHiT-Illumina for rapid response samples
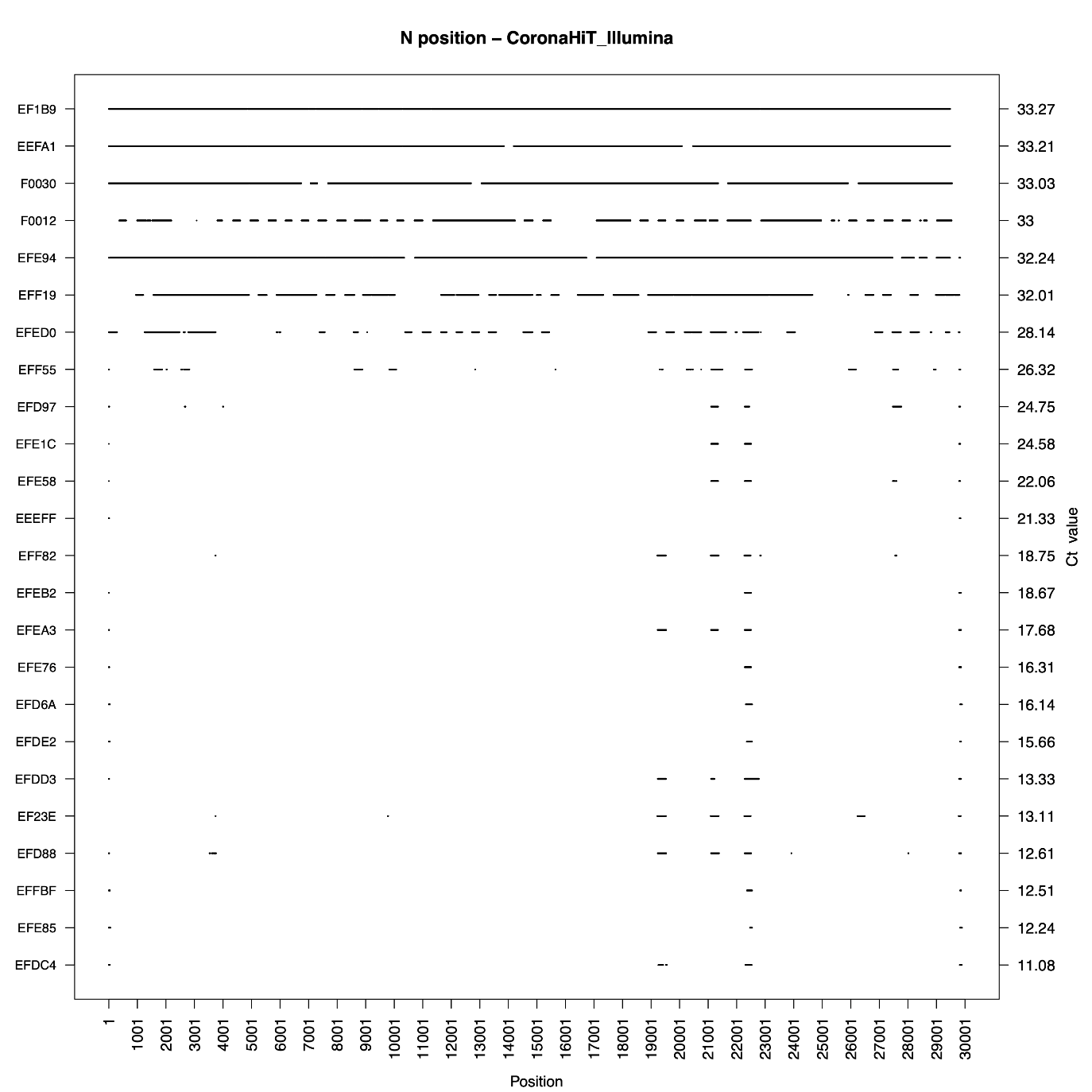

Supplement: Supplementary file 2 — Additional file 2: Figures S1-S6. Overview of SARS-CoV-2 genome coverage drop-out regions (Ns) of each sample that had a known Ct, for the individual sequencing experiments: Figure S1. ARTIC LoCost for routine samples. Figure S2. CoronaHiT-ONT for routine samples. Figure S3 CoronaHiT-Illumina for routine samples. Figure S4. ARTIC LoCost for rapid response samples. Figure S5. CoronaHiT-ONT for rapid response samples. Figure S6. CoronaHiT-Illumina for rapid response samples. [file 13073_2021_839_MOESM2_ESM.docx]
